# Factors affecting antiretroviral pharmacokinetics in HIV-infected women with virologic suppression on combination antiretroviral therapy: a cross-sectional study

**DOI:** 10.1186/1471-2334-13-256

**Published:** 2013-06-03

**Authors:** Mona Rafik Loutfy, Sharon Lynn Walmsley, Marina Barbara Klein, Janet Raboud, Alice Lin-in Tseng, Sandra Lauren Blitz, Neora Pick, Brian Conway, Jonathan Benjamin Angel, Anita Rochelle Rachlis, Kevin Gough, Jeff Cohen, David Haase, David Burdge, Fiona Mary Smaill, Alexandra de Pokomandy, Hugues Loemba, Sylvie Trottier, Charles Jean la Porte

**Affiliations:** 1 Women’s College Research Institute, Women’s College Hospital, 790 Bay Street, Room 736, Toronto, Ontario M5G 2N8, Canada; 2 Department of Medicine, University of Toronto, Toronto, Ontario, Canada; 3 University Health Network, Toronto, Ontario, Canada; 4 McGill University Health Centre, Montreal Chest Institute, Montreal, Quebec, Canada; 5 Dalla Lana School of Public Health, University of Toronto, Toronto, Ontario, Canada; 6 Department of Medicine, University of Toronto, Toronto, Ontario, Canada; 7 Oak Tree Clinic, BC Women’s Hospital and Health Centre, Vancouver, British Columbia, Canada; 8 Department of Medicine, University of British Columbia, Vancouver, British Columbia, Canada; 9 Department of Pharmacology and Therapeutics, University of British Columbia, Vancouver, British Columbia, Canada; 10 Ottawa Hospital Research Institute, Toronto, Ontario, Canada; 11 Department of Medicine, University of Ottawa, Toronto, Ontario, Canada; 12 Sunnybrook Health Sciences Centre, Toronto, Ontario, Canada; 13 Department of Medicine, St. Michael’s Hospital, Toronto, Ontario, Canada; 14 Windsor Regional Hospital Metropolitan Campus, Windsor, Ontario, Canada; 15 Department of Medicine, Division of Infectious Diseases, Dalhousie University, Halifax, Nova Scotia, Canada; 16 Victoria General Hospital, Halifax, Nova Scotia, Canada; 17 Faculty of Medicine, Division of Infectious Diseases, University of British Columbia, Vancouver, British Columbia, Canada; 18 Department of Pathology and Molecular Medicine, McMaster University, Hamilton, Ontario, Canada; 19 Centre Hospitalier De L’Universite De Montreal, Hopital Notre-Dame, Montreal, Québec, Canada; 20 University of Ottawa Health Services, Ottawa, Ontario, Canada; 21 Centre Hospitalier Universitaire de Quebec – pavillon CHUL, Quebec, Quebec, Canada

**Keywords:** HIV, Women, Antiretroviral therapy, Pharmacokinetics

## Abstract

**Background:**

Although some studies show higher antiretroviral concentrations in women compared to men, data are limited. We conducted a cross-sectional study of HIV-positive women to determine if protease inhibitor (PI) and non-nucleoside reverse transcriptase inhibitor (NNRTI) C_min_ and C_max_ values were significantly different than historical general population (predominantly male) averages and to evaluate correlates of higher concentrations.

**Methods:**

HIV-positive women with virologic suppression (viral load < 50copies/mL) on their first antiretroviral regimen were enrolled. Timed blood samples for C_min_ and C_max_ were drawn weekly for 3 weeks. The ratio of each individual’s median C_min_ and C_max_ to the published population mean values for their PI or NNRTI was calculated and assessed using Wilcoxon sign-rank. Intra- and inter-patient variability of antiretroviral drug levels was assessed using coefficient of variation and intra-class correlation. Linear regression was used to identify correlates of the square root-transformed C_min_ and C_max_ ratios.

**Results:**

Data from 82 women were analyzed. Their median age was 41 years (IQR=36-48) and duration of antiretrovirals was 20 months (IQR=9-45). Median antiretroviral C_min_ and C_max_ ratios were 1.21 (IQR=0.72-1.89, p=0.003) (highest ratios for nevirapine and lopinavir) and 0.82 (IQR=0.59-1.14, p=0.004), respectively. Nevirapine and efavirenz showed the least and unboosted atazanavir showed the most intra- and inter-patient variability. Higher CD4+ count correlated with higher C_min_. No significant correlates for C_max_ were found.

**Conclusions:**

Compared to historical control data, C_min_ in the women enrolled was significantly higher whereas C_max_ was significantly lower. Antiretroviral C_min_ ratios were highly variable within and between participants. There were no clinically relevant correlates of drug concentrations.

**Trial registration:**

NCT00433979

## Background

Since the advent of combination antiretroviral therapy (cART), there has been a dramatic decrease in the mortality of individuals infected with human immunodeficiency virus (HIV) [1]. Traditionally, this combination has included two drugs from the antiretroviral class of nucleoside reverse transcriptase inhibitors (NRTIs) and either one from the class of protease inhibitors (PIs) or one from the class of non-nucleoside reverse transcriptase inhibitors (NNRTIs) [2]. If the third agent is from the PI class, it is often combined with a low dose of ritonavir in order to boost its drug levels [2]. More recently, raltegavir has been added as an option for the third agent, but remains rarely used in Canada due to cost [3].

Despite these tremendous advances in HIV management, there remain several important complications related to antiretroviral drug use, one of the most notable being drug-related adverse events (AEs) and toxicities [4], which can negatively impact patients’ quality of life, contributing to non-adherence and drug resistance and ultimately effectiveness. Adverse drug reactions to antiretrovirals are a major reason for discontinuing or changing therapy [5,6]. Furthermore, these drug-related complications can significantly contribute to morbidity, hospitalizations, and mortality in this population [7-10].

Women constitute one of the fastest-rising population groups at risk for infection with HIV, representing over 50% of cases worldwide, and approximately 25% of new cases in the United States (U.S.) and 28% of new cases in Canada [1-3]. Surprisingly, little is known about the differential efficacy and toxicity of various antiretroviral drugs in women compared to men [11]. This gap in knowledge is a result of the initial exclusion and continued underrepresentation of women in antiretroviral clinical trials [12]. This circumstance has slowly started to change, and there are now more longitudinal studies examining women-specific issues [13,14]. Many studies in the general population have shown that AEs are more common in women than in men [15]. In the HIV-infected population, higher incidence rates of increased systemic symptoms (such as nausea, vomiting and diarrhea), as well as organ toxicity (including anemia, hepatotoxicity, pancreatitis, lactic acidosis, peripheral neuropathy, and notable lipodystrophy), have been observed in women compared to men [11,16,17]. For nevirapine, female gender and higher CD4+ cell counts were risk factors for fatal hepatitis, and this observation has led regulatory authorities to release warnings on its use in certain female populations [18].

Most of these studies assessing sex differences in antiretroviral AEs rates are limited in that they only identify the issue of increased toxicity in women and do not try to elucidate the cause or management [16,19,20]. The potential causes of these gender differences in antiretroviral toxicities may have a sound biologic basis possibly related to differences in physiology and/or the influence of sex hormones on drug metabolism. All aspects of drug handling and exposure may be different in women versus men, including bioavailability (with lower gastric emptying time due to hormonal contraception use or pregnancy) and distribution (lower body weight, smaller organ size, higher body fat content, altered gastric motility, greater organ blood flow, and altered protein binding secondary to endogenous or exogenous estrogens). Metabolism and elimination of drugs have also exhibited gender differences related to differences in expression and activity of various drug transporters and metabolizing enzymes [20,21]. Of particular interest, a better understanding of antiretroviral pharmacokinetics (PK) in women and how these drug levels impact AEs and toxicities in women is crucial, and will lead to more effective methods of treatment, reduced discomfort, possible enhanced adherence and improved morbidity and/or mortality.

Although a few investigations have considered PK differences that occur between men and women, most of these studies involved the use of older antiretroviral agents and were assessed in small sample sizes [21-29]. Furthermore, a number of potentially confounding variables such as race, age, weight, menstruation, and hepatitis co-infection have not been explored as determinants impacting drug levels in a female population. We conducted a cross-sectional study of HIV-positive women taking their first combination ART to determine if drug levels (C_min_ and C_max_) of currently used PIs and NNRTIs were significantly higher in this population as compared to the historical general (predominantly male) population and to evaluate correlates of higher concentrations.

## Methods

### Ethics statement

The study was reviewed and received ethics approval by the Full Institutional Research Ethics Board (REB) of main coordinating research centre, Women’s College Research Institute, Toronto, Canada (REB# 2006–003). Additional Full Institutional REB approval was obtained from each research site prior to commencement. All study personnel were trained in and practiced under the principles of the Declaration of Helsinki. All study candidates were informed about this study and written informed consent was obtained from every participant prior enrollment.

### Study population

We carried out a cross-sectional study with participants who met the following inclusion criteria: 1) HIV-positive, 2) biologically female, 3) 18 years of age or older, 4) taking their first cART regimen containing either a PI or an NNRTI with a backbone of NRTI as per common practice for at least 3 months (but could have had prior switches that were not due to virologic failure), 5) taking either a PI or an NNRTI but not both, 6) if taking a PI, must have been taking only one PI excluding low dose ritonavir used as boosting and 7) had evidence of full virologic suppression (HIV-1 RNA VL < 50 copies/mL) on at least two occasions at least one month apart. The patient population was limited to women who were on their first cART regimen in order to ensure a more homogeneous population. The current analysis was limited to candidates who were taking the following antiretroviral drugs (atazanavir, atazanavir boosted with ritonavir, lopinavir boosted with ritonavir, efavirenz and nevirapine) [3,30]. Participants could not take both a PI and NNRTI or two PIs excluding ritonavir as there are multiple drug interactions between these agents which would lead to uninterpretable results. Further, participating women had to have full virologic suppression to avoid inclusion of women who experience difficulty with drug adherence. The 3 month requirement for being on a cART regimen was mandated to 1) ensure stabilization of adherence as there may be an adjustment period as patients get accustomed to taking their new drug regimen, 2) eliminate early discontinuations due to drug toxicities which often happen during the first 3 months of therapy, and to a lesser extend 3) to ensure drug steady state as there is some variation between drugs [5].

A planned sample size of 80 was calculated in order to estimate if the mean ratio of the C_min_ values to historical values for the general population was significantly different from 1.0, assuming an alternative hypothesis of 1.2 with a standard deviation of 0.64, 80% power and a significance level of 0.05. Recruitment was conducted from February, 2007 to November, 2008 from 14 primary care and specialty HIV clinics from across Canada. Recruitment and study qualification determination was carried out by the site investigator and research staff. The recruitment was carried out in a non-random consecutive manner as research staff were instructed to invite every consecutive qualifying woman who received services in their clinic on all days that care was provided.

### Data collection

Study visits occurred at weekly intervals for a three-week period. During the baseline visit, demographic and HIV and other medical history data was collected including concurrent medications, validated questionnaires pertaining to antiretroviral adherence [31] and symptom distress [32] were completed, weight was measured and blood work to assess laboratory values was drawn.

### Pharmacokinetic analysis

At each of the three visits, patients had a pre-dose (C_min_) and a maximum (C_max_) PI or NNRTI drug level drawn. Patients were asked to fast from midnight of the previous night. One hour before their scheduled morning dose the pre-dose blood collection was drawn (C_min_ t) (alternative arrangements were made if the patient took her drugs in the evening). Following the pre-dose blood collection, patients consumed a standard breakfast (consisting of 50% carbohydrate, 30% fat, 20% protein) after which time the morning antiretroviral dose was administered. C_max_ levels were drawn at 2 hours post-dose for atazanavir 400 mg QD [33], 3 hours post-dose for atazanavir/ritonavir 300/100 mg QD [33], 4 hours post-dose for lopinavir/ritonavir 400/100 mg BID [34], 5 hours post-dose for efavirenz 600 mg QD [35], and 2 hours post-dose for nevirapine (both 200 mg BID and 400 mg QD [36]). The plasma drug level samples were all stored in cryovials at −20°C or lower and shipped for concurrent assessment at the end of the study. Concentrations of the PIs and the NNRTIs in plasma were measured simultaneously by sensitive and selective, validated high-performance liquid chromatography coupled to tandem mass-spectroscopy (LC-MS/MS) [37]. All samples were analyzed at the pharmacokinetic laboratory at the Ottawa Hospital Research Institute in Ottawa, Canada.

The PK endpoints for each patient were determined by taking the median of the three weekly values for the C_min_ and C_max._ The rationale for measuring drug levels at three separate time points was that there is potentially intra-individual variability in drug levels, especially for PIs [38,39]. Using the median value for the endpoints eliminated outliers. The three drug level measurements allowed for the calculation of intra-patient variability.

### Statistical analyses

Baseline characteristics of the study population were summarized using medians and interquartile ranges (IQR) for continuous variables and frequencies and proportions for categorical variables.

The mean C_min_ and C_max_ drug levels in the historical HIV population were taken from the most recent product monograph when possible, or from a published study if the product monograph did not report these values [33-36]. Next, for each subject the ratio of their median C_min_ and C_max_ for their main PI or NNRTI to this published drug level was calculated and used as the primary outcome as previously reported by Burger et al. [40] Due to the lack of published population PK data for ritonavir when included as part of regimen in a “boosting” role, we only calculated the ratios for the main PI in a participant’s regimen. Differences between the C_min_ ratio and C_max_ ratio to the population mean were conducted using a Wilcoxon sign-rank test for a median of 1. For comparison purposes, PK levels were dichotomized into high and low levels with a high level defined as ≥ 1.5 X arithmetic population mean of the C_min_ and C_max_ for each drug. Inter-patient variability of C_min_ and C_max_ for each antiretroviral drug was assessed by calculating the coefficient of variation (CV) using each individual’s median C_min_ and C_max_. An intra-patient CV for each participant was calculated from the C_min_ and C_max_ values obtained at each of the 3 visits. These values are summarized using median and IQR of the individual CVs. The inter-patient CV is a measure of variation among individuals whereas intra-patient CV measures variation within an individual The inter-patient CV is calculated as the ratio of the standard deviation to the mean X 100; the higher the value, the more variability exists. Finally, linear regression models were fit to assess correlates of the square root-transformed C_min_ and C_max_ ratios. The square-root transformation was used because the untransformed C_min_ and C_max_ ratios were both right-skewed. The regression models included indicator variables to account for the different antiretroviral medications and each potential correlate was included separately in an adjusted model. Statistical analyses were performed using SAS Version 9.2 (SAS Institute, Cary, North Carolina, USA).

## Results

### Study population

Ninety women were enrolled from 14 sites across Canada between 2/2007 and 11/2008. Eight women were excluded for the following reasons: one due to missing data, six were not on a current cART regimen deemed eligible to be included in this analysis, and one because she was not on standard dosing schedule. The data is summarized for the remaining 82 patients. Median age of the study population was 41 years (IQR 36–48) and 56% identified as Black. The median times since their HIV diagnosis and start of their current cART regimen were 7 years (IQR 3–11) and 20 months (IQR 9–45), respectively. Fifty-seven percent were taking a PI-containing regimen (81% being ritonavir-boosted and 19% unboosted) and 43% a NNRTI-containing regimen. All participants had an undetectable viral load with a median CD4+ count at the time of enrolment of 487 cells/μL (IQR 380–621). Additional demographic and clinical variables of the study population are summarized in Table 1. No relevant concurrent medications were being taken by the participants.

**Table 1:** Demographic characteristics of study participants

***Characteristics***	***Total n=82***
Age	41 (36–48)
Race	
White	28 (34%)
Black	46 (56%)
Other	8 (10%)
Risk Factor	
Injection drug use	11 (13%)
Endemic country	27 (33%)
Heterosexual contact	59 (72%)
Blood transfusion	7 (9%)
Unknown	8 (10%)
Years since HIV diagnosis	7 (3–11)
CD4+ cell count prior to cART (μL)	232 (128–400)
Current CD4+ cell count (μL)	487 (380–621)
VL prior to cART (log_10_ copies/mL)	4.5 (3.0-5.0)
AIDS diagnosis	19 (23%)
Months since start of cART	20 (9–45)
cART includes PI	47 (57%)
cART includes NNRTI	35 (43%)
Missed ARV dose in past week	6 (7%)
Hepatitis B co-infection	2 (2%)
Hepatitis C co-infection	10 (12%)
Weight (kg)	67.3 (60.3-81.5)
BMI	25.8 (22.4-31.3)
Menstrual status	
Regular periods	44 (54%)
Irregular periods	10 (12%)
Current amenorrhea	9 (11%)
Menopausal	19 (23%)

Continuous variables presented as medians with interquartile range; categorical variables presented as n (%).*VL*, viral load; *AIDS*, acquired immunodeficiency syndrome; *cART*, combination antiretroviral therapy; *PI*, protease inhibitor; *NNRTI*, non-nucleoside reverse transcriptase inhibitor; *ARV*, antiretroviral; *BMI*, body mass index.

### Drug level ratios by antiretroviral drug

**Figure 1:**
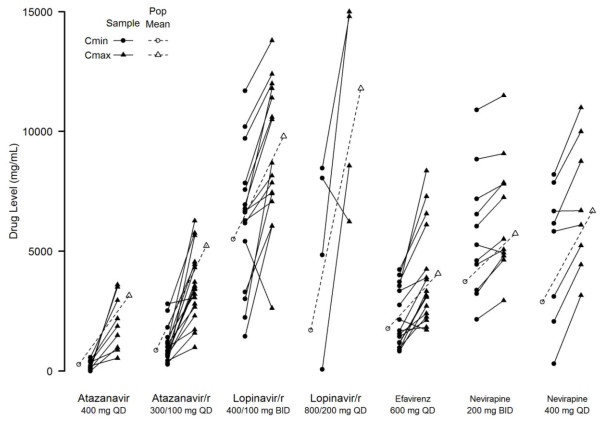
**Paired C**_**min **_**and C**_**max **_**values for each participant by antiretroviral drug and dose.** The C_min_ values are identified by circles and the C_max_ values by triangles. The reported population mean C_min_ and C_max_ values for each antiretroviral drug and dose are presented with the hatched lines (−−−).

The C_min_ and C_max_ summaries and historical values used to calculate the ratios are reported in Table 2[33-36]. Paired values for the C_min_ and C_max_ data for each dosing regime are shown in Figure 1. Overall, the median ratio of the participants’ C_min_ to historical mean values was 1.21 (IQR 0.72-1.89, p=0.003) and the median ratio of C_max_ to historical mean values was 0.82 (IQR 0.59-1.14, p=0.004). Twenty-eight participants (34%) had C_min_ ≥ 1.5 X arithmetic population mean of the C_min_ for each drug and seven participants (9%) had C_max_ ≥ 1.5 X arithmetic population mean of the C_max_ for each drug; all on an NNRTI. The median ratios of C_min_ and C_max_ to historical mean values for each specific antiretroviral drug overall and for each dose are presented in Table 3.

**Table 2:** **Reference population mean and Study Participants C**_**min **_**and C**_**max **_**by antiretroviral drug**

					***C***_***min***_		***C***_***max***_	
Antiretroviral agent*	***Reference***	***Dose (mg)***	***Freq***	***N***	***Population mean (ug/mL)***	***Study participants median (IQR)***	***Population mean (ug/mL)***	***Study participants median (IQR)***
Atazanavir**	[33]	400	QD	9	273	214 (95–373)	3152	1870 (979–2950)
Atazanavir** boosted with ritonavir	[33]	300	QD	18	862	835 (663–1220)	5233	3430 (2670–4450)
Lopinavir boosted with ritonavir	[34]	400	BID	16	5500	6660 (4360–7710)	9800	8420 (7240–11600)
Lopinavir boosted with ritonavir	[34]	800	QD	4	1700	6445 (2452–8260)	11800	11685 (7403–14900)
Efavirenz	[35]	600	QD	16	1768	1680 (1180–3450)	4072	3235 (2330–5180)
Nevirapine	[36]	200	BID	11	3730	5270 (3380–7190)	5740	5510 (4810–7860)
Nevirapine	[36]	400	QD	8	2880	5995 (2590–7275)	6690	6400 (4838–9380)

*QD*, once daily; *BID*, twice daily; *IQR*, interquartile range.

*Nucleos(t)ide backbone: of 9 participants on Atazanavir 400 mg QD, all 9 were taking Abacavir/3TC; of 18 participants on Atazanavir/ritonavir 300 mg/100 mg QD, 11 were taking Abacavir/3TC, 3 Tenofivir/FTC, 3 Tenofivir/3TC and 1 Zidovudine/3TC; of 16 participants on Lopinavir/ritonavir 400 mg/100 mg BID, 7 were taking Zidovudine/3TC, 6 Abacavir/3TC, 1 Tenofivir/FTC, 1 Tenofivir/3TC and 1 was on PI monotherapy; of 4 participants on Lopinavir/ritonavir 800 mg/200 mg QD, 2 were taking Tenofivir/FTC, 1 Abacavir/3TC, 1 Didanosine/3TC; of 16 participants on Efavirenz 600 mg QD, 5 were taking Tenofivir/FTC, 5 Abacavir/3TC, 5 Zidovudine/3TC, 1 Tenofovir/3TC; of 11 participants on Nevirapine 200 mg BID, 7 were taking Zidovudine/3TC, 4 Abacavir/3TC; of 8 participants on Nevirapine 400 mg/ QD, 4 were taking Abacavir/3TC, 1 Tenofivir/FTC, 1 Zidovudine/3TC, 1 Tenofivir/3TC, 1 Didanosine/3TC.

** Of the 27 particpants taking Atazanavir or Atazanvir/ritonavir, none were taking any gastric acid suppression medication.

**Table 3:** **Ratio of C**_**min **_**and C**_**max **_**values to Historical Population Means by antiretroviral drug and dose**

			**C**_**min**_			**C**_**max**_		
**Antiretroviral agent**		***n***	***n (%) >1.5 historical pop Mean***	***Media ratio to historical population mean median ratio (IQR)***	***p-value***^***a***^	***n (%) >1.5 Historical pop mean***	***Media ratio to historical population mean Median ratio ( IQR)***	***p-value***^***a***^
All		82	28 (34.1%)	1.21 (0.72-1.89)	<.01	7 (8.5%)	0.82 (0.59-1.14)	<.01
Atazanavir	400 QD	9	2 (22.2%)	0.78 (0.35-1.37)	0.71	0 (0.0%)	0.59 (0.31-0.94)	0.04
Atazanavir (boosted with ritonavir)	300 QD	18	4 (22.2%)	0.97 (0.77-1.42)	0.58	0 (0.0%)	0.66 (0.51-0.85)	<.001
All Atazanavir		27	6 (22.2%)	0.95 (0.53-1.42)	0.76	0 (0.0%)	0.65 (0.44-0.87)	<.0001
Lopinavir (boosted with ritonavir)	400 BID	16	3 (18.8%)	1.21 (0.79-1.40)	0.27	0 (0.0%)	0.86 (0.74-1.18)	0.32
Lopinavir (boosted with ritonavir)	800 QD	4	3 (75.0%)	3.79 (1.44-4.86)	0.25	0 (0.0%)	0.99 (0.63-1.26)	0.63
All Lopinavir		20	6 (30.0%)	1.22 (0.79-1.81)	0.11	0 (0.0%)	0.86 (0.72-1.21)	0.29
Efavirenz	600 DQ	16	6 (37.5%)	0.95 (0.67-1.95)	0.37	4 (25.0%)	0.79 (0.57-1.27)	0.63
Nevirapine	200 BID	11	5 (45.5%)	1.41 (0.91-1.93)	0.03	2 (18.2%)	0.96 (0.84-1.37)	0.46
Nevirapine	400 QD	8	5 (62.5%)	2.08 (0.90-2.53)	0.08	1 (12.5%)	0.96 (0.72-1.40)	0.95
All Nevirapine		19	10 (52.6%)	1.62 (0.91-2.32)	<0.01	3 (15.8%)	0.96 (0.81-1.37)	0.47

*IQR*, Interquartile range. ^a^ Sign rank test for a median ratio different than 1.

### Intra-patient and inter-patient variability of antiretroviral drug levels

The intra- and inter-patient variability of each antiretroviral drug’s C_min_ and C_max_ was assessed using CV; data are presented in Table 4. Data from 2 participants for whom PK samples were only obtained at one visit were excluded from the intra-patient variability calculations. Nevirapine BID and lopinavir/ritonavir BID showed the least inter-patient variability with the lowest CVs for both C_min_ and C_max_. Nevirapine BID and efavirenz had the lowest intra-patient variability variability for Cmin, while nevirapine BID, lopinavir/ritonavir BID, and efavirenz demonstrated lowest intra-patient variability for Cmax. Inter- and intra- patient variability was high for atazanavir, particularly when not boosted with ritonavir.

**Table 4:** **Inter-patient and Intra-patient variability of C**_**min **_**and C**_**max **_**for each antiretroviral agent**

			**C**_**min**_			**C**_**max**_		
**Antiretroviral agent**		***n***	***Mean (SD)***	***Inter-patient CV***	***Intra-patient CV median (IQR)***	***Mean (SD)***	***Inter-patient CV***	***Intra-patient CV median (IQR)***
Atazanavir	400 QD	9	250.4 (195.4)	78.0%	57.4 (40.5-86.7)	2000.4 (1145.5)	57.3%	61.9 (44.2-66.0)
Atazanavir (boosted with ritonavir)	300 QD	18	1065.6 (699.2)	65.6%	26.3 (15.6-56.1)	3525.3 (1459.1)	41.4%	32.7 (17.2-54.5)
Lopinavir (boosted with ritonavir)	400 BID	16	6370.0 (2846.4)	44.7%	23.8 (12.8-75.8)	8991.3 (2953.3)	32.8%	14.0 (12.3-21.7)
Lopinavir (boosted with ritonavir)	800 QD	4	5356.2 (3882.3)	72.5%	63.4 (35.2- 115)	11151.3 (4433.2)	39.8%	15.6 ( 7.4-28.4)
Efavirenz	600 QD	16	2196.9 (1209.2)	55.0%	17.7 (10.5-25.1)	3929.4 (2060.6)	52.4%	15.5 (12.8-27.9)
Nevirapine	200 BID	11	5693.6 (2586.8)	45.4%	13.9 (10.2-27.8)	6490.9 (2454.9)	37.8%	10.7 ( 7.8-19.6)
Nevirapine	400 QD	8	5027.9 (2867.2)	57.0%	21.3 (12.1-44.9)	6924.4 (2761.1)	39.9%	22.8 ( 8.3-53.8)

*SD*, Standard deviation; *CV*, Coefficient of Variation; *IQR*, Interquartile Range.

### Linear regression models for antiretroviral C_min_ and C_max_

Linear regression models to assess the relationship between the square root-transformed C_min_ ratio and demographic and clinical variables of the population were carried out (Table 5). Higher CD4+ cell count was the only variable significantly associated with higher C_min_ ratios (Beta coefficient=0.04/ 100 cells per μL increase, 95% CI=0.003-0.080; p=0.03). Similar analyses were carried out for the assessment of the correlates of the square root-transformed C_max_ ratios and only injection drug use showed a trend to be negatively associated with C_max_ ratio (Beta coefficient=−0.10, 95% CI=−0.23-0.02; p=0.10).

**Table 5:** **Linear regression models for square-root transformed C**_**min **_**and C**_**max**_

	**C**_**min**_			**C**_**max**_		
***Variables***	***Beta***	***95% CI***	***p-value***	***Beta***	***95% CI***	***p-value***
Age (per 10 years)	0.02	(−0.07 - 0.11)	0.62	0.00	(−0.04 - 0.05)	0.95
Race						
White	Reference					
Black	−0.16	(−0.35 - 0.03)	0.09	−0.06	(−0.16 - 0.04)	0.25
Other	−0.20	(−0.50 - 0.11)	0.20	0.01	(−0.15 - 0.17)	0.91
Risk Factor						
IDU	−0.21	(−0.45 - 0.04)	0.10	−0.10	(−0.23 - 0.02)	0.10
Endemic country	−0.08	(−0.26 - 0.10)	0.38	−0.07	(−0.16 - 0.02)	0.14
Heterosexual contact	0.13	(−0.05 - 0.32)	0.16	0.07	(−0.03 - 0.16)	0.16
Blood transfusion	−0.08	(−0.38 - 0.22)	0.60	−0.08	(−0.23 - 0.07)	0.31
Unknown	−0.06	(−0.34 - 0.23)	0.69	0.01	(−0.14 - 0.15)	0.90
Years since HIV diagnosis (per 10 years)	0.00	(−0.00 - 0.00)	0.84	−0.00	(−0.00 - 0.00)	0.53
Baseline CD4 (per 100/μL)	0.02	(−0.02 - 0.06)	0.28	0.00	(−0.02 - 0.02)	0.97
Baseline CD4 > 200/μL	0.07	(−0.11 - 0.24)	0.46	0.03	(−0.06 - 0.12)	0.51
Current CD4 (per 100/μL)	0.04	( 0.00 - 0.08)	0.03	0.01	(−0.01 - 0.03)	0.61
Current CD4 > 200/μL	0.30	(−0.25 - 0.84)	0.28	0.16	(−0.12 - 0.44)	0.26
Baseline VL (log_10_ copies/mL)	−0.02	(−0.09 - 0.06)	0.64	−0.00	(−0.04 - 0.03)	0.81
AIDS diagnosis	0.07	(−0.12 - 0.27)	0.46	0.06	(−0.04 - 0.16)	0.27
Years on current regimen	−0.01	(−0.05 - 0.03)	0.68	−0.00	(−0.02 - 0.02)	0.83
Hepatitis B co-infection	0.22	(−0.34 - 0.78)	0.44	−0.04	(−0.33 - 0.24)	0.76
Hepatitis C co-infection	0.18	(−0.07 - 0.44)	0.17	−0.10	(−0.23 - 0.03)	0.14
Smoking Status						
Smoker	Reference					
Previous Smoker	0.02	(−0.27 - 0.31)	0.89	0.05	(−0.10 - 0.20)	0.49
Never	−0.13	(−0.31 - 0.06)	0.18	0.02	(−0.08 - 0.11)	0.70
Hypertension	0.14	(−0.12 - 0.41)	0.28	0.02	(−0.12 - 0.15)	0.82
Diabetes	0.02	(−0.43 - 0.47)	0.94	−0.05	(−0.28 - 0.18)	0.66
Weight (per kg)	0.00	(−0.00 - 0.01)	0.36	0.00	(−0.00 - 0.00)	0.32
BMI (per kg/m^2^)	0.00	(−0.01 - 0.02)	0.47	0.00	(−0.00 - 0.01)	0.64
Menopausal (self-reported)	0.05	(−0.14 - 0.25)	0.59	0.03	(−0.07 - 0.14)	0.55

*IDU*, injection drug use; *VL*, viral load; *AIDS*, acquired immunodeficiency syndrome; *BMI*, body mass index.

## Discussion

In this cross-sectional study of 82 HIV-positive women taking cART with full virologic suppression, median pre-dose drug levels were found to be significantly higher than historical controls. Approximately one- third of women had a C_min_ value more than 1.5 times higher than historical control values. Since the majority of historical controls consist of men, this finding supports the growing literature indicating that drug levels are higher in women than in men.

Our findings also support the increasing body of literature on the gender differences in drug disposition and PK of all drugs. However, in the HIV population, these differences may in part be due to race as many of the historical controlled participants were White men, and as in our study, many of the females infected with HIV globally are Black. Several studies have investigated the relationship of antiretroviral PK with genetically determined factors that might differ by individual ancestral history [41-45]. Racial differences have been found in P-glycoprotein (PGP) activity, an efflux protein which pumps its substrates, including the PIs, out of cells, away from their site of action. For example, Africans are four times more likely than white Americans or Japanese individuals to have the CC genotype at position 3435 of the gene coding for PGP [45]. Fellay and colleagues [41] demonstrated that persons with the CC genotype have higher PGP-activity, higher serum drug levels. Similarly, there are racial differences in the prevalence of slow-metabolizers of specific pathways, for example 2D6 and 2B6, of the cytochrome P-450 system of drug metabolism in the liver [43]. Thus, there may be genetically determined heterogeneity in the *in vivo* transport and metabolism of antiretroviral agents, resulting in variation in serum and intracellular drug levels.

A number of other studies have assessed the gender differences in antiretroviral PK, particularly of the older PIs. In a study of 186 patients (15.6% female), Fletcher and colleagues [23] demonstrated that serum levels of saquinavir were significantly higher in women than in men, independent of body size. Another study sought to characterize the PK of saquinavir (1000 mg BID), lopinavir (400 mg BID), and ritonavir (100 mg BID) in a multidrug rescue therapy study [24]. Twenty-five patients (28% women) were included in the study group and fifteen (20% women) were included in the comparison group that did not receive saquinavir. Area under the curve (AUC), C_max_, and C_min_ values for saquinavir and ritonavir were significantly higher in women than in men, though there were no significant differences in weight or body mass indexes between genders. Work by Dickinson and colleagues, who looked at the PK of saquinavir and ritonavir in 34 patients on this combination, showed that in women a higher exposure to saquinavir might, at least in part, be driven by higher exposure to ritonavir [25]. Pharmacokinetic studies of newer PIs have only been reported in product monographs limited to data showing that women have modest increases in AUC of approximately 20% for lopinavir, atazanavir and darunavir [33,34,46]. In terms of NNRTIs, investigators have demonstrated higher serum efavirenz and nevirapine levels in women [26-29]. A previous report using a full 12 hour PK of nevirapine showed a gender difference of an 18.9% lower AUC in males when corrected for body weight [26]. In the same study, pregnant women had lower nevirapine exposure and this effect did not seem to be driven by body weight.

Our data also adds to the literature on gender differences in C_max_ of current antiretroviral agents. These observations should be interpreted with caution as the collection of C_max_ samples in this study was done at a single standardized timepoint; therefore, if the C_max_ sampling time was slightly off from the real T_max_ then the resulting C_max_ observations would be interpreted as lower. In our study, atazanavir had the lowest ratios of both C_min_ and C_max_ versus historical reference values, but also the greatest observed inter-patient variability. In general, a higher degree of variability was noted for C_min_ as compared to C_max_ values.

Few studies have assessed intra-patient variability of antiretroviral drug levels for either gender. Over our 3-week sampling period, we found higher intra-patient variability in the PI-based regimens compared to the NNRTIs; a similar finding to the retrospective summary provided by Fabbiani and colleagues [47]. More specifically, unboosted atazanavir had the highest intra- and inter-patient CVs for C_min_ and C_max_, whereas efavirenz and nevirapine had the lowest CVs for intra-patient variability. This most likely reflects the longer half-life for both nevirapine and efavirenz, compared to PIs. Despite ritonavir boosting for both, the intra-patient variability for lopinavir C_max_ was substantially lower than that of atazanavir, which may reflect the absence of a food and gastric pH effects on lopinavir absorption as compared to atazanavir absorption. In addition, lopinavir is co-formulated with ritonavir, thus ensuring simultaneous coadministration of both drugs, whereas atazanavir absorption may be impacted if ritonavir is not taken at the same time for reasons such as patient choice (e.g., concerns of selective side effects of ritonavir) or forgetfulness since ritonavir capsules require refrigeration. In our study, inter-patient CV for C_min_ and C_max_ of boosted and unboosted atazanavir appeared to be lower than historical values from predominantly male populations, while intrapatient CV for C_min_ of unboosted atazanavir appeared to be higher than historical data. For NNRTIs, observed intra- and inter-patient C_min_ CVs of efavirenz and nevirapine appeared to be lower than historical controls. As such, the existence of sex-based differences in antiretroviral variability cannot be ruled out.

Correlates of antiretroviral drug levels in women have only been investigated by few studies. Gibbons and colleagues examined the potential for age-dependent changes in lopinavir and efavirenz levels in female subjects in a retrospective analysis of therapeutic drug monitoring on non-pregnant women receiving either lopinavir/ritonavir 400 mg /100 mg twice daily or efavirenz 600 mg daily [48]. They found that women > 50 years of age had significantly higher 8–16 hour efavirenz levels when compared to women < 40 years of age (p=0.046). In our study, we found that C_min_ ratio increased with CD4+ cell count and there was some indication that it was lower among injection drug users. There were no statistically significant correlates of C_max_. Of note, there was no correlation between drug levels and body weight or body mass index. The lack of statistical significance may be related to the attempts to make our population homogenous.

Our study has a number of limitations, most notably the lack of a concurrent male control group which would have allowed real-time assessment of sex differences in drug levels. The fact that historical control data was used results in the inability to report on the demographics of the controls including the true proportion of cases that were male, ethnicity, weight, and co-infection status. The restriction of our study to women on their first cART regimen with virologic suppression to ensure homogeneity likely contributed to the lack of range amongst our covariates, decreasing our ability to detect differences and associations. If higher concentrations were associated with toxicity, then the women may have switched off the regimen or been inconsistently adherent and experienced viral failure and would not have been eligible for this data set. The demand and time commitment for the participants was high and likely led to some degree of selection bias of women who are committed to therapy and research. Also, there were small sample sizes for each drug dosing (e.g. only 4 participations taking lopinavir/ritonavir 800 mg/100 mg OD and 8 taking nevirapine 400 mg OD) and for this reason it was difficult to make conclusions regarding specific drugs and drug dosing.

## Conclusion

In summary, our data adds to the growing literature on the gender differences of antiretroviral drug levels. Our study showed that the PIs and NNRTIs overall C_min_ ratios were significantly higher in our HIV-positive female participants as compared to men (historical controls). In particular, we observed the highest C_min_ for nevirapine and lopinavir. These latter observations add to the literature by providing data on newer antiretroviral agents, and could explain nevirapine’s important gender-specific drug toxicity. Our study was also able to study intra- and inter-patient variability for PIs and NNRTIs C_min_ ratios and found significant variability particularly for unboosted and boosted atazanavir. This intra-patient variability could have clinical implications with respect to toxicity and efficacy. These findings require further study to elucidate the mechanism and clinical consequences of these differences and results. It also emphasizes the importance of gender-specific analyses when investigating antiretroviral efficacy and toxicity.

## Abbreviations

AEs: Adverse events; AIDS: Acquired immune deficiency syndrome; ART: Antiretroviral therapy; AUC: Area under the curve; BID: Twice daily; BMI: Body mass index; cART: Combination antiretroviral therapy; CHUL: Centre Hospitalier Universitaire de Quebec; CI: Confidence interval; CIHR: Canadian Institutes of Health Research; Cmax: Maximum plasma concentration of the drug; Cmin: Minimum plasma concentration of the drug; CV: Coefficient variation; CVs: Coefficient variations; FRSQ: Fonds de recherche en santé du Québec; HIV: Human immunodeficiency virus; IDU: Injection drug user; IQR: Interquartile range; NNRTI: Non nucleoside reverse transcriptase inhibitor; NNRTIs: Non nucleoside reverse transcriptase inhibitors; PGP: P-glycoprotein; PI: Protease inhibitor; PIs: Protease inhibitors; PK: Pharmacokinetics; QD: Once daily; REB: Research ethics board; TDM: Therapeutic drug monitoring; VL: Viral load

## Competing interests

There are no financial or non-financial competing interests related to this paper and project.

## Authors’ contributions

MRL was the senior most responsible investigator for the project who had study idea, wrote the protocol, sought funding and coordinated the project. MRL wrote the first and final drafts of the manuscript and is the corresponding author. CJlP and ALT provided pharmacokinetic expertise to the project and provided substantive edits to the manuscript. CJlP carried out the PK testing of all the samples. SLB conducted the statistical analyses under the direction of JMR, MRL and CJlP. SLB and JMR provided substantive edits to the manuscript. SB assisted with referencing. SW, MBK, NP, BC, JBA, ARR, KG, JC, DH, DB, FMS, AdP, HL, ST contributed to the study idea and protocol, acted as site investigators and enrolled participants for the study, and contributed to editing the manuscript. All authors reviewed the manuscript during preparation, provided critical feedback and approved the final manuscript.

## Pre-publication history

The pre-publication history for this paper can be accessed here:

http://www.biomedcentral.com/1471-2334/13/256/prepub
